# Other Hospitals at Which Clinical Instruction Is Given

**Published:** 1898-09-03

**Authors:** 


### OTHER HOSPITALS AT WHICH CLINICAL INSTRUCTION IS GIVEN.

In addition to the regular medical schools mentioned above, many hospitals offer opportunities for clinical instruction, and students will do well in their fifth year to widen their clinical experience by resorting to some of them. It should be noted that several of the special hospitals have instituted more or less complete courses of lectures on their special subjects.

It is especially desirable that students should obtain a good knowledge of those fevers which are excluded from the wards at general hospitals. For this purpose the hospitals of the Metropolitan Asylums Board are available. Apply at the offices, Norfolk Street, Strand.

The following are a few of the hospitals in London open for clinical work:—

General Hospitals.

Great Northern Central Hospital.

London Temperance Hospital.

Seamen's Hospitals: The Dreadnought Hospital, Greenwich, and the Branch Hospital, Victoria and Albert Docks.

West London Hospital, Hammersmith Road.

Children's Hospitals.

Hospital for Sick Children, Great Ormond Street.

East London Hospital for Children, Shadwell.

North-Eastern Hospital for Children, Hackney Road, Shoreditch.

Ophthalmic Hospitals.

Royal London Ophthalmic Hospital, Moorfields.

Royal Westminster Ophthalmic Hospital, King William Street, Strand.

Royal Eye Hospital, Southwark.

Diseases of the Chest.

Hospital for Consumption and Diseases of the Chest, Brompton.

City of London Hospital for Diseases of the Chest, Victoria Park.

Other Special Hospitals.

Bethlem Royal Hospital.

National Hospital for the Paralysed and Epileptic, Queen's Square.

Central London Throat and Ear Hospital, Gray's Inn Road.

St. Peter's Hospital for Stone and Urinary Diseases, Covent Garden.

St. John's Hospital for Diseases of the Skin, 49, Leicester Square.

